# Systemic colonization of clover (*Trifolium repens*) by *Clostridium botulinum* strain 2301

**DOI:** 10.3389/fmicb.2015.01207

**Published:** 2015-10-31

**Authors:** Matthias Zeiller, Michael Rothballer, Azuka N. Iwobi, Helge Böhnel, Frank Gessler, Anton Hartmann, Michael Schmid

**Affiliations:** ^1^Research Unit Microbe-Plant Interactions, Department for Environmental Sciences, German Research Center for Environmental Health – Helmholtz Zentrum MünchenNeuherberg, Germany; ^2^Bavarian Health and Food Safety AuthorityOberschleißheim, Germany; ^3^Institute for Applied Biotechnology in the Tropics at the Georg-August University GoettingenGoettingen, Germany; ^4^miprolab GmbHGoettingen, Germany

**Keywords:** *Clostridium botulinum*, plant-associated-pathogenic-endophytic-bacteria, FISH, CLSM, diagnostic PCR, plant-growth-promotion, botulinum-neurotoxin D (BoNT D)

## Abstract

In recent years, cases of botulism in cattle and other farm animals and also in farmers increased dramatically. It was proposed, that these cases could be affiliated with the spreading of compost or other organic manures contaminated with *Clostridium botulinum* spores on farm land. Thus, soils and fodder plants and finally farm animals could be contaminated. Therefore, the colonization behavior and interaction of the botulinum neurotoxin (BoNT D) producing *C. botulinum* strain 2301 and the non-toxin producing *Clostridium sporogenes* strain 1739 were investigated on clover (*Trifolium repens*) in a field experiment as well as in phytochamber experiments applying axenic and additionally soil based systems under controlled conditions. Plants were harvested and divided into root and shoot parts for further DNA isolation and polymerase chain reaction (PCR) assays; subsamples were fixed for fluorescence *in situ* hybridization analysis in combination with confocal laser scanning microscopy. In addition, we observed significant differences in the growth behavior of clover plants when inoculated with clostridial spores, indicating a plant growth promoting effect. Inoculated plants showed an increased growth index (shoot size, wet and dry weight) and an enlarged root system induced by the systemic colonization of clover by *C. botulinum* strain 2301. To target *C. botulinum* and *C. sporogenes*, 16S rDNA directed primers were used and to specifically detect *C. botulinum*, BoNT D toxin genes targeted primers, using a multiplex PCR approach, were applied. Our results demonstrate an effective colonization of roots and shoots of clover by *C. botulinum* strain 2301 and *C. sporogenes* strain 1739. Detailed analysis of colonization behavior showed that *C. botulinum* can occur as individual cells, in cell clusters and in microcolonies within the rhizosphere, lateral roots and within the roots tissue of clover.

## Introduction

Members of the genus *Clostridium*, which contain a broad spectrum of Gram-positive, obligate anaerobic, rod-shaped and spore-forming bacteria, are ubiquitously distributed in soil, water, decaying organic matter, and other ecological niches. Some of these bacteria produce potent neurotoxins which are responsible for neurological diseases in humans and animals, like tetanus or botulism (Böhnel et al., 2001; Johnson and Bradshaw, 2001). On the other hand, many clostridial strains are widely used in biotechnology for the production of organic solvents like acetone, ethanol, butanol (Jang et al., 2012) or they occur also in methane producing anaerobic fermentation plants (Morris, 1989). A common feature within the genus is the ability to form endospores during nutrient deficient conditions rendering them extremely resistant against many adverse environmental factors (Andreesen et al., 1989). *Clostridium botulinum* strains are defined by the production of various types of neurotoxins, but this production is not correlated with the *C. botulinum* phenotypes and also non-toxigenic variants exist (Lindström et al., 2001). Phylogenetically closely related *Clostridia* which are not able to produce a botulinum neurotoxin are taxonomically assigned to various types such as *Clostridium subterminale* or *C. sporogenes. Clostridium botulinum* is well-known for the production of the highly potent and so far most toxic biological substance known. Botulinum Neurotoxin (BoNT) causes a disease called botulism with a parenteral lethal dose for BoNT A of 1 ng/kg body weight for human beings (Gill, 1982). Currently, eight distinct groups of *C. botulinum* neurotoxins, related to the eight serotypes of BoNTs (A-H) that vary serologically, are known with a variety of subtypes (Smith and Sugiyama, 1988; Hill et al., 2007; Dover et al., 2014). In group III the toxin gene could be described as a mixture of different fragments of BoNT C and D and representing a variation of the toxin gene cluster (BoNT C-D, D-C: toxin chimeras, mosaic toxin genes), which differs from typical toxin of type C and D strains (Moriishi et al., 1996). The botulinum neurotoxin can be genetically encoded on the chromosome or on different genetic elements like plasmids or by bacteriophages, depending on the strain type or toxin type. Strains assigned as *C. botulinum* are extremely heterogeneous in their physiological properties. Four genetically distinct lineages within the *C. botulinum* complex exist (Collins and East, 1998). These organisms can be separated into four phenotypic groups (I–IV) because of their physiological properties. The occurrence of the different groups depends on temperature, water activity, pH value, and nutrient supply. Soil is considered to be the major habitat of proteolytic group I organisms producing toxin types A, B, and F (Smith and Sugiyama, 1988). The occurrence of non-proteolytic group II strains, producing toxin types E, B and F, is closely associated with aquatic ecosystems with high water activity (Hauschild and Dodds, 1993), whereas the main reservoirs for group III *Clostridia*, producing toxin types C and D, are soils and sediments which are in close contact with water and the gastrointestinal tract of animals (Smith and Sugiyama, 1988). Type G was found in soil of a field in Argentina (Giménez and Ciccarelli, 1970) and type H was isolated from a baby with infant botulism (Barash and Arnon, 2014).

The rhizosphere is a hot spot with extraordinary significance for soil microbial communities and for microbe–plant interactions. Root exudates provide a large variety of easily degradable organic carbon sources that attract microbes and support the growth of the best adapted populations, which results in selection of a highly active microflora in the rhizosphere of plants (Hartmann et al., 2008). The rhizosphere microbiome with its highly diverse physiological activities, contributes significantly to sustainable plant growth. In addition to symbiotic plant microbe systems, a great diversity of plant-associated soil bacteria with more or less close association are able to promote the growth of a wide range of economically important crops by competing with plant and human/animal pathogens for the colonization of plant surfaces and contributing to enhanced nutrient uptake (Berg et al., 2005; Bashan et al., 2014).

Endophytic colonization of plants by bacteria and fungi is well-documented and in contrast to the colonization by plant pathogens, it does not harm plants and may even improve their development and health (Rosenblueth and Martínez-Romero, 2006). However, plants (e.g. lettuce) can also be colonized systemically by human pathogenic bacteria and this contamination may not always be removed by washing (Hofmann et al., 2014). Thus, endemic bacterial colonization by human/animal pathogens could facilitate the transmission of these strains from the field via the food production chain to the consumer with severe impact on the health of animals and human beings.

Although *Clostridia* are known to be distributed in soils and other ecological niches, plants as hosts for epi- or endophytic colonization have not been investigated so far. Only a few studies showed that diverse clostridial strains can indeed colonize plants (Minamisawa et al., 2004; Miyamoto et al., 2004; Ye et al., 2005; Timmers et al., 2012) and several anaerobic nitrogen-fixing consortia (ANFICO) were described consisting of nitrogen-fixing *Clostridia* and non-diazotrophic bacteria in gramineous plants (Minamisawa et al., 2004; Miyamoto et al., 2004; Ye et al., 2005). Minamisawa et al. (2004) phylogenetically analyzed 40 anaerobic N_2_-fixing isolates derived from various plant species (*Oryza* sp., *Miscanthus sinensis, Saccharum spontaneum*, or *Polygonum sachalinense*) from different countries. ANFICOs are widespread in wild rice species and pioneer plants. Isolates could be affiliated to cluster I and XVIa among the 17 clusters within the genus *Clostridium* defined by Collins et al. (1994). Additionally, all tested isolates could utilize a variety of sugars as carbon source such as glucose, cellobiose and mannose, indicating the affiliation to saccharolytic *Clostridia*, which is often a feature of plant associated microbes. Miyamoto et al. (2004) described novel endophytic nitrogen-fixing *Clostridia* in the grass *M. sinensis*, like *Clostridium saccharoperbutylacetonicum, Clostridium roseum, Clostridium acetobutylicum*, and *Clostridium beijerinckii*. In their study, the *Clostridia* belonged exclusively to group II of cluster XIVa and groups IV and V of cluster I (based on Collins classification scheme) and could further be detected in roots and aerial parts of plants, like stems and leaves, where they build a large proportion of the diazotrophic bacterial community in *M. sinensis*. Although *Clostridia* typically exhibit an obligate anaerobic lifestyle, the studies of Minamisawa et al. (2004) and Miyamoto et al. (2004) conclusively indicate that they colonize and grow successfully in plant tissues and may thus have some tolerance to oxygen.

Biogas production which employs anaerobic fermentation of sustainable substrates (Oleskowicz-Popiel et al., 2012) like corn and grain or also biological waste or farm manure becomes more and more important nowadays as a CO_2_ neutral alternative energy source for climate protection (Kleerebezem and van Loosdrecht, 2007). Via agricultural practices in terms of organic farming like application of compost (Böhnel and Lube, 2000), manure or slurry, as well as post-processed residues of anaerobic fermentation of organic waste as organic fertilizers on fields, pathogens and/or toxin producing bacteria like *Clostridia* could be introduced into soil and subsequently colonize the rhizosphere of crop plants (Böhnel and Lube, 2000). A burden and enrichment of these microorganisms could result in contamination of any kind of crops which are subsequently harvested to feed animals (corn, barley, oat, grass, and clover) or man (lettuce, spinach, and tomatoes) and provide therefore a risk for humans and animals.

Botulism is a worldwide occurring severe and fatal disease affecting humans and animals which is characterized by flaccid paralysis of muscles due to the inhibition of acetylcholine release at the neuromuscular junction caused by BoNT (Smith and Sugiyama, 1988; Böhnel and Gessler, 2005). In general, group I and II of *C. botulinum* cause different forms of botulism in humans: Botulism can occur as a classical food borne intoxication, or as an infection, like in infant botulism (Pickett et al., 1976) and similarly in adults (“hidden botulism”; Chia et al., 1986). Most cases of botulism in animals are caused by organisms of group III because Type C strains are dangerous for poultry (ducks, geese and chicken) and Type D strains for mammals (horses, cattle, sheep, pigs, dogs, deer, and mink). In many countries, e.g., Brazil, serotypes C and D are responsible for causing botulism in cattle (Döbereiner et al., 1992). Outbreaks of botulism have also been reported in Europe (Böhnel et al., 2005; Payne et al., 2011) and North America (Livesey et al., 2004) and there is also great concern that infected animals will become a source of human food-borne botulism. In recent years, a continuous increase of botulism outbreaks mainly in cattle was observed (Böhnel and Lube, 2000; Payne et al., 2011). A possible cause for these findings may be the contamination of fields by *C. botulinum* spores and the consumption of microbiologically contaminated feed.

In an earlier field study (Gessler and Böhnel, 2006), it has been shown that if compost spiked with *C. botulinum* spores (10^3^ and 10^5^ spores g^-1^ compost) was added to botulinum free agricultural soil, *C. botulinum* was still detectable after 757 and 939 days at considerable densities. Since an interaction with clover plants was obvious because a striking plant growth stimulation effect was visible in these plots, in depth interaction studies were initiated on the colonization of white clover (*Trifolium repens*) by the neurotoxin D producing strain *C. botulinum* 2301.

## Materials and Methods

### Bacterial Strains, Culture Conditions, and Spore Production

*Clostridium sporogenes* (1739), *C. botulinum* (IBT 2301; Institute of Biotechnology, Culture Collection of Switzerland [CCOS] of ZHAW) and *Rhizobium leguminosarum* (DSM 6039) were used in this study. *C. sporogenes* 1739 is a non-toxigenic strain and *C. botulinum* 2301 is a toxigenic type D strain. *R. leguminosarum* DSM 6039 was grown using the DSMZ medium No. 98 at 30°C with moderate shaking and was inoculated to all laboratory growth systems to ensure the formation of active nodules. For solid media 15 g l^-1^ agar was added.

For all *Clostridium* strains reinforced clostridial medium (RCM; Oxoid, Wesel, Germany) was used: yeast extract, 3 g l^-1^; meat extract, 10 g l^-1^; peptone, 10 g l^-1^; D-glucose, 5 g l^-1^; sodium chloride, 5 g l^-1^; soluble starch, 1 g l^-1^; sodium acetate, 3 g l^-1^; cysteine hydrochloride, 0.5 g l^-1^; agar, 0.5 g l^-1^; resazurin, 2.5 mg l^-1^. The pH was adjusted to 6.8 ± 0.2 with NaOH (1 M). The medium was aliquoted into anaerobic serum flasks (Glasgeraetebau Ochs, Bovenden, Germany) and autoclaved under anaerobic conditions following standard procedures for generating anaerobic media (Hungate, 1969). The serum flasks were incubated at 37°C without shaking. For plate assays solid RCM medium (15 g l^-1^ agar) in petri dishes with specialized vents were used in anaerobic jars (Oxoid, Wesel, Germany) containing resazurin as an oxygen indicator. Inoculations of cultures were carried out under anaerobic conditions in an anaerobic chamber or with a very short time of exposure to air.

For spore production of *Clostridia* a stock culture was grown anaerobically in RCM medium at 37°C for 48 h. 20% (v/v) of this preculture was inoculated into cooked meat medium (CMM: heart muscle, 454 g l^-1^; peptone, 10 g l^-1^; ‘Lab-Lemco’ powder, 10 g l^-1^; NaCl, 5 g l^-1^; D-glucose, 2 g l^-1^; resazurin, 2.5 mg l^-1^; pH 7.2 ± 0.2) followed by anaerobic incubation at 37°C. As soon as sporulation rate was about 80–100% within 6–7 days, spores were harvested by centrifugation and repeatedly washed with phosphate-buffered saline (PBS) and subjected to sonication to avoid clumping of spores. The toxin producing *C. botulinum* strain 2301 was cultivated additionally in 10 1 clostridial medium (Gessler and Böhnel, 2006). Samples were monitored daily for contamination by plating on RCM medium (Oxoid, Wesel, Germany). The degree of sporulation was quantified microscopically.

### Surface Disinfection and Germination of Clover (*Trifolium repens*) Seeds

Clover Seeds were obtained from Herbiseed (Twyford, UK). To eliminate contamination of microorganisms, seeds were treated with 1% (v/v) Tween 80 and surface sterilized with 70% (v/v) ethanol and sodium hypochlorite solution (2–4% active Cl) according to standard protocols (Rothballer et al., 2008). After five washing steps in sterile deionized water a treatment with antibiotics (Penicillin 0.6 mg ml^-1^; Streptomycin 0.25 mg ml^-1^) for 30 min was performed. After incubating the seeds for 3–4 days at room temperature (18°C) in the dark on NB medium agar plates (meat extract, 1 g l^-1^; peptone, 5 g l^-1^; yeast extract, 2 g l^-1^; pH 7.1 ± 0.2; Sigma-Aldrich; Taufkirchen, Deutschland) to allow germination of seeds and emergence of roots until they reach a length of at least 1 cm, sprouts without any visible contamination were washed to remove remaining antibiotic solution and selected for further inoculation experiments.

### Plant Growth Studies

To determine possible plant growth promoting effects of inoculated *Clostridia*, white clover (*T. repens*) was used as plant model system. For inoculation, clostridial spores (10^5^–10^7^ spores ml^-1^) were mixed with *R. leguminosarum* (1 × 10^7^ CFU ml^-1^) in PBS and 1 ml of this solution was added directly on the base of each plant seedling by pipetting.

### Mono-axenic Inoculations

Sterile Phytatray^TM^ boxes (Sigma-Aldrich) were filled with washed and autoclaved quartz sand (particle size 3 mm) together with 25 ml MS-Medium (Murashige and Skoog, 1962). Surface sterilized, germinated seedlings were planted into the substrate and inoculated as described above. Boxes were placed in a phytochamber (BioLine VB1514, Voetsch Industrietechnik, Reiskirchen, Germany) and incubated under controlled environmental conditions [14 h light (23°C)/10 h dark (18°C), 50% relative humidity] for about 4 weeks. After growth period, plants were carefully harvested under sterile conditions and separated in roots and shoots. Adherent particles of sand were washed away with sterile PBS solution.

### Inoculation in Soil Systems

For cultivation of clover commercially available soil composed of natural clay, peat and sod peat containing abundant nutrients (ED-73, Bayerische Gaertnereigenossenschaft, Aschheim, Germany) was used. The soil was thoroughly sieved and potted into plant trays with 5 cm × 5 cm dimension. After wetting, surface sterilized, germinated seedlings were planted and inoculated as described above. As negative control, *R. leguminosarum* without *Clostridium* was inoculated. To test the system for sterility, 2–3 plant replicates were treated with 1 ml of sterile PBS only. The pots were placed in a phytochamber as described above for Phytatray^TM^ boxes.

### Biomass Determination in Field Experiment

Spiking of compost, spreading on plots and seeding were performed as it was described previously (Gessler and Böhnel, 2006). On day 727 (approximately at the end of year two) plant samples of all plots were taken to estimate plant growth and biomass. A square frame with an inner side length of 30 cm was randomly placed on the plots in triplicates. Plants were harvested and collected. Grass was manually separated from clover in the laboratory. The dry weight of each fraction was determined by drying the fractions at 105°C overnight.

### Quantification of *C. botulinum* Strain 2301 Spores in Field Experiment

Soil samples of the root zone were obtained as previously described (Muratova et al., 2003). At day 727 after inoculation, clover root samples were taken from a plot on which a high *C. botulinum* strain 2301 concentration had been added during the experiment (Gessler and Böhnel, 2006). Bulk soil was recovered from the samples and the clover roots were weighed and placed in Erlenmeyer flasks containing 50 ml of PBS. The flasks were shaken for 30 min at 175 rpm. Roots were transferred to a new flask containing the same amount of PBS and again shaken for 30 min. Thereafter flasks were treated with high-frequency ultrasound in an ultrasonic bath (35 kHz) for 1 min to remove still adhering bacteria from root surfaces. Roots were then dried and weighed to determine the root biomass. The suspensions of both flasks were centrifuged at 4000 × *g* for 15 min. The supernatants were discarded and the pellet was resuspended in PBS in a total volume of 10 ml. Bacteria collected in the first flask were considered to be rhizosphere organisms, in the second flask rhizoplane bacteria. Spores were counted in bulk and rhizosphere soil as well as on the rhizoplane using a previously published two-tube Most-Probable Number-PCR (MPN-PCR; Gessler and Böhnel, 2006).

### Statistical Analysis

Statistical analyses were performed using the GraphPad Prism software (GraphPad Software, Inc., San Diego, CA, USA). The obtained data (shoot size, shoot fresh weight, and shoot dry weight) were not transformed logarithmically (log10) before statistical analysis. The data were analyzed using students *t*-test and significance (*p* < 0.05) was calculated. Data were checked to be normally distributed and samples are assumed to be independent. For calculation of the differences of shoot size and fresh and dry weight of plants the *t*-test was used to compare the differently treated plants against each other. There were at least 17 plants per batch used for the determination of statistical differences in the growth of plants.

### DNA Isolation

High molecular weight DNA from bacterial cultures and plant tissues were isolated using the FastDNA SPIN kit for Soil (Bio101, MP Biomedicals, Heidelberg, Germany) according to manufacturer’s instructions. For DNA isolation of pure cultures overnight grown *Clostridia* cells were used. In addition, DNA isolation from plant tissues was performed after harvesting and removing adhering soil particles. The roots and shoots were homogenized thoroughly using liquid nitrogen. *Clostridia* were selectively enriched in RCM to allow spores to germinate and grow as vegetative cells before DNA isolation.

### PCR Detection

The polymerase chain reaction (PCR) based detection method can be used for the rapid diagnosis of botulism caused by *C. botulinum* type D. Two different diagnostic PCR systems were applied to test for the presence of *C. botulinum* DNA. The primer pair P930/P932 (Le Bourhis and Saunier, 2005) targets the 16S-rDNA and was specific for the genus *Clostridium*. The primer pair DS11/DS22 (Takeshi et al., 1996) was specific for the toxigenic *C. botulinum* type D strains targeting a fragment of the BoNT D gene. In addition a BoNT/CD multiplex PCR system was used as a method for distinguishing between the different toxin chimeras (BoNT C/CD/DC/D) caused by mosaic toxin genes. Type-specific primer sets for detecting C and D toxin genes were designed based on the toxin gene sequences reported previously. For multiplex PCR two specific primer pairs IBTSB F2 (5′-TGGTTCACCTTTTATGGGAGA-3′)/IBTSB R2 (5′-TGTACGTTGGGTCCATCTTG-3′) and IBTSB F4 (5′-TCAGATGCTTTGGATTAGAGATTT-3′)/IBTSB R4 (5′-ACTTCAAAGGATTTCCCCAAT-3′) were used. All primers used in this study were purchased from Eurofins Medigenomix (Ebersberg, Germany). PCR assays were performed in a total reaction volume of 50 μl. The reaction mix contained PCR/Taq buffer, MgCl_2_ (2.5 mM), dNTPs (200 mM each), primers (50 μM of each), Taq polymerase (0.5 U; Qiagen, Hilden, Germany) and 10 ng template DNA of the respective sample. PCR was performed in a programmable thermal cycler (Primus, MWG Biotech, Ebersberg, Germany). The PCR procedure starts with an initial denaturation at 94°C for 5 min, followed by 30–35 cycles of 94°C for 1 min, annealing at 55–60°C for 1 min and elongation at 72°C for 30 s and additionally a final elongation at 72°C for 10 min. To analyze the sizes of the amplification products, 10 μl of the PCR products were subjected to horizontal gel electrophoresis in 2.0% agarose and ethidium bromide staining according to standard techniques. The different subtypes of toxins could be distinguished by amplification product size. The PCR products on agarose gels of type D *C. botulinum* toxigenic strains were 665, 497, 462, and 128 bp, respectively (**Figure 5**).

### Sensitivity of PCR

Template DNA solutions obtained from *C. botulinum* type D were adjusted to 5.0 ng/ml in a spectrophotometer (Nanodrop, Thermo Fisher Scientific, Waltham, MA, USA). After a serial dilution down to 1 pg/ml, each preparation was amplified by PCR using four primer sets and their products were detected by ethidium bromide staining after electrophoresis.

### FISH-CLSM Studies

Root samples were fixed in 4% paraformaldehyde and 50% (v/v) ethanol (Roller et al., 1994) and subjected to fluorescence *in situ* hybridization (FISH), using a modified version of the previously published 16S-rRNA phylogenetic oligonucleotide probe Chis150 (Franks et al., 1998; TTATGCGGTATTAATCTCCCTTT) specific for clostridial strains in combination with probe mix EUB338-I,-II,-III (Amann et al., 1990; Daims et al., 1999) specific for the domain *Bacteria*. Clostridial cells of subcluster I show a yellow fluorescence signal in the overlay of RGB images of recorded channels of the confocal laser scanning microscopy (CLSM) after successful hybridization of both probes; *R. leguminosarum* shows fluorescence in green due to the exclusive binding of the general bacterial probe. The third fluorescence channel (Cy5) shows the structure of plant tissue by autofluorescence. All probes used in this study were purchased from Eurofins Medigenomix.

Fixed samples were cut into 50–100 μm thick lateral sections with a razor blade. For preparing longitudinal slices, roots were cut into halves. The slices were transferred to eight-well adhesive teflon-coated slides (Paul Marienfeld, Bad Mergentheim, Germany) and were air dried at 50°C. The hybridizations were carried out with 35% formamide in the hybridization buffer for stringent conditions as it was previously described (Amann et al., 1992; Manz et al., 1996). A three dimensional localization of bacterial cells within the plant samples was performed using a CLSM (LSM-510 META, Zeiss, Oberkochen, Germany) equipped with an argon laser (488 nm for fluorescein) using a BP 500–550 filter and two helium-neon lasers (543 nm for Cy3 and 633 nm for Cy5) using LP 560 and LP 650 filters. The fluorescence of fluorescein and Cy3 are depicted in green and red, respectively, while Cy5 is assigned a blue color. In general only two fluorescent dyes were used at the same time and the third channel was used to visualize the autofluorescence of plant cell walls.

## Results

### Plant Growth Stimulation Effect – Field Experiment

**FIGURE 1:**
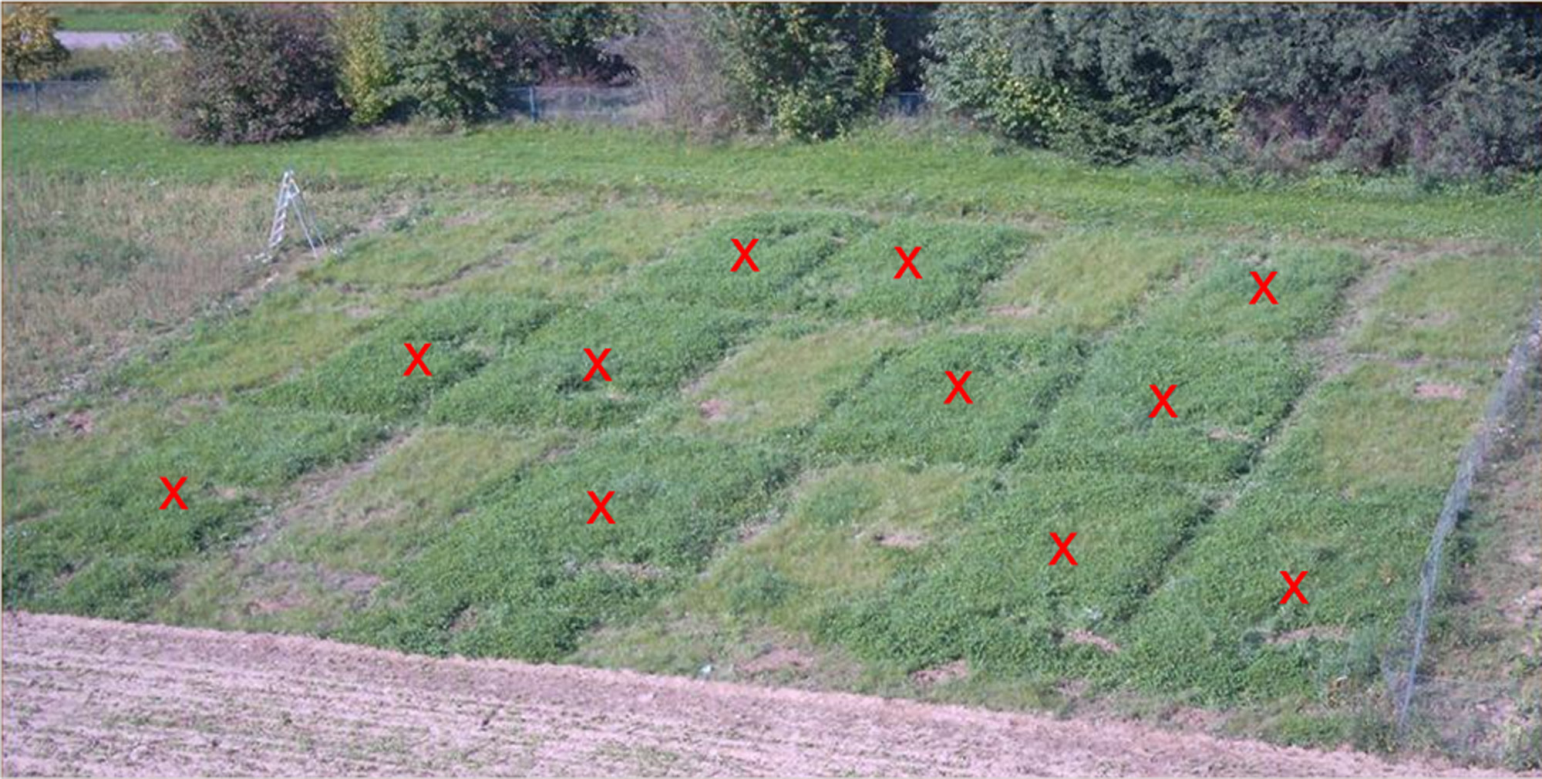
**Experimental plots of the field experiment.** Enhanced clover growth can be seen on the plots in darker green (red crosses). These plots received compost spiked with *Clostridium botulinum* strain 2301 spores. The other plots were treated with compost free of *C. botulinum* or served as a negative control with no compost treatment.

**FIGURE 2:**
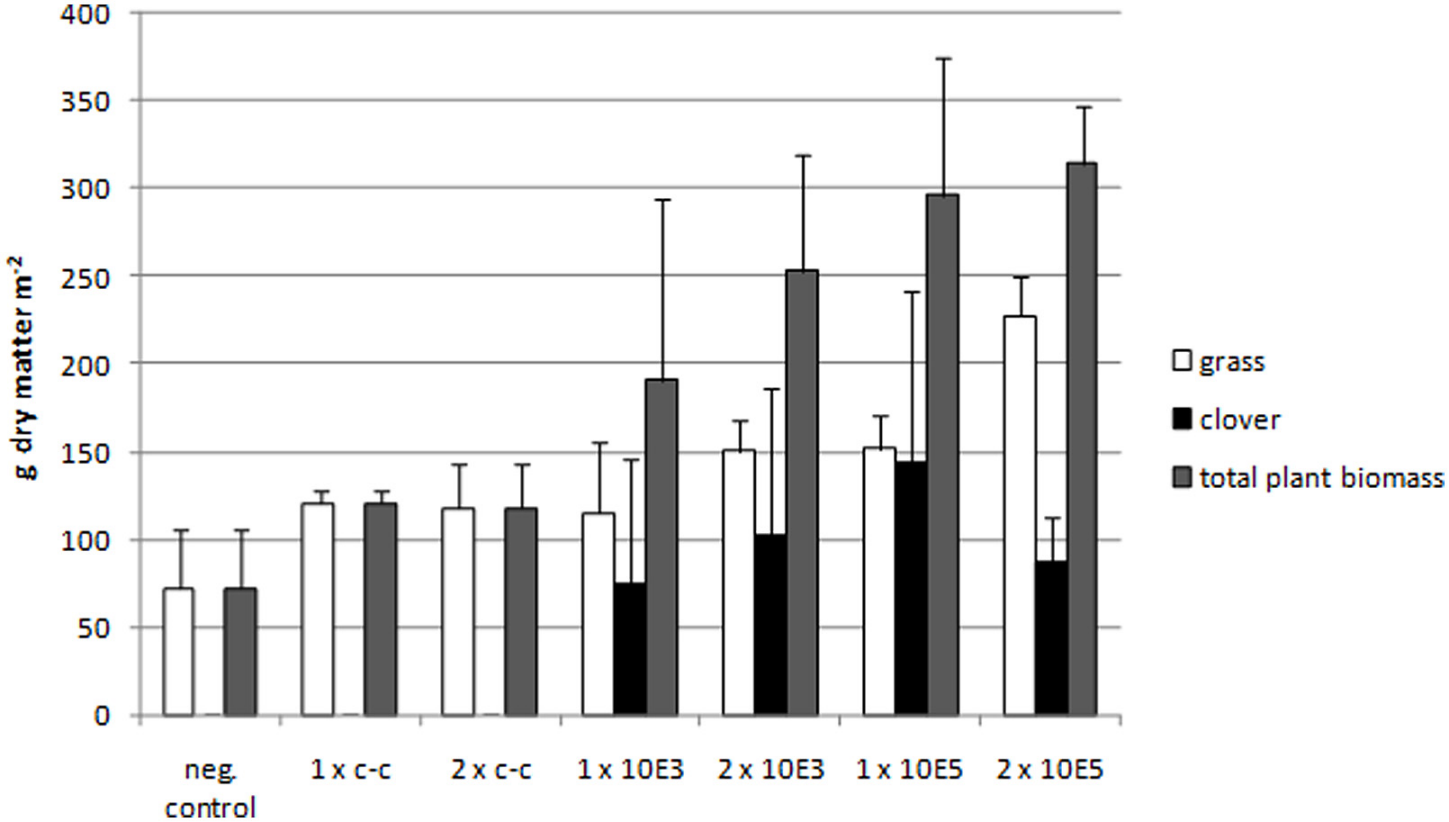
**Measurements of clover, grass, and total biomass dry matter of the field experiment in the second year.** An increase of total biomass depending on the amount of spores applied can be seen. Negative control: no compost amendment; 1xc-c: compost control, one application; 2xc-c: compost control, two applications; 1x10E3: compost with 10^3^ *C. botulinum* spores g^-1^, one application; 2x10E3: compost with 10^3^ *C. botulinum* spores g^-1^, two applications; 1x10E5: compost with 10^5^ *C. botulinum* spores g^-1^, one application; 2x10E5: compost with 10^5^ *C. botulinum* spores g^-1^, two applications. From each treatment (with botulinum spores in the two concentrations and the compost and negative control) we had four replicates in the field trial (*N* = 4). On each plot we randomly placed a square frame (30 cm length) three times and collected all plants (above soil) within the frame. Clover and grass were separated manually. We determined the dry matter clover/grass for each plot individually. The values plotted in the graph show the arithmetic mean of the four respective plots and the standard deviation. Negative control: Grass and total plant biomass: Mean ± SEM: 72,215 ± 34,225. 1xc-c: Grass and total plant biomass: Mean ± SEM: 120,544 ± 7,070. 2xc-c: Grass and total plant biomass: Mean ± SEM: 117,766 ± 25,897. 1x10E3: Grass: Mean ± SEM: 115,544 ± 39,763. 1x10E3: Clover: Mean ± SEM: 75,178 ± 71,003. 1x10E3: Total plant biomass: Mean ± SEM: 190,722 ± 103,462. 2x10E3: Grass: Mean ± SEM: 150,726 ± 17,787. 2x10E3: Clover: Mean ± SEM: 102,212 ± 83,886. 2x10E3: Total plant biomass: Mean ± SEM: 252,938 ± 66,124. 1x10E5: Grass: Mean ± SEM: 151,837 ± 19,157. 1x10E5: Clover: Mean ± SEM: 144,43 ± 79,591. 1x10E5: Total plant biomass: Mean ± SEM: 296,267 ± 78,444. 2x10E5: Grass: Mean ± SEM: 227,385 ± 22,450. 2x10E5: Clover: Mean ± SEM: 87,028 ± 26,322. 2x10E5: Total plant biomass: Mean ± SEM: 314,413 ± 32,826.

A striking plant growth promoting effect on clover was observed in plots of the field experiment (Gessler and Böhnel, 2006), where *C. botulinum* strain 2301 spores were applied (**Figure 1**). Clover growth was only observed in plots treated with *C. botulinum* spores (**Figure 2**); a correlation between the amount of *C. botulinum* spores introduced into soil and the amount of grown biomass of clover was observed in the second year, except for plots that were amended twice with the highest spore concentration (**Figure 2**). In this treatment an increase in grass biomass by 2.6 times compared to the controls was measured, following the trend in total biomass increase (**Figure 2**). This effect was reflected by the increase in biomass and was sustained at least throughout the following year (data not shown).

Soil samples of the plant rhizosphere were collected. In bulk soil 20 *C. botulinum* spores per gram of soil were detected, while 5 × 10^4^ spores per gram of rhizosphere soil and 2 × 10^2^ per gram wet weight of clover roots were counted. These data indicate an increase in the number of spores associated with clover roots.

### Growth Chamber Pot Experiment

To investigate these findings further, growth chamber experiments were performed under defined conditions.

**FIGURE 3:**
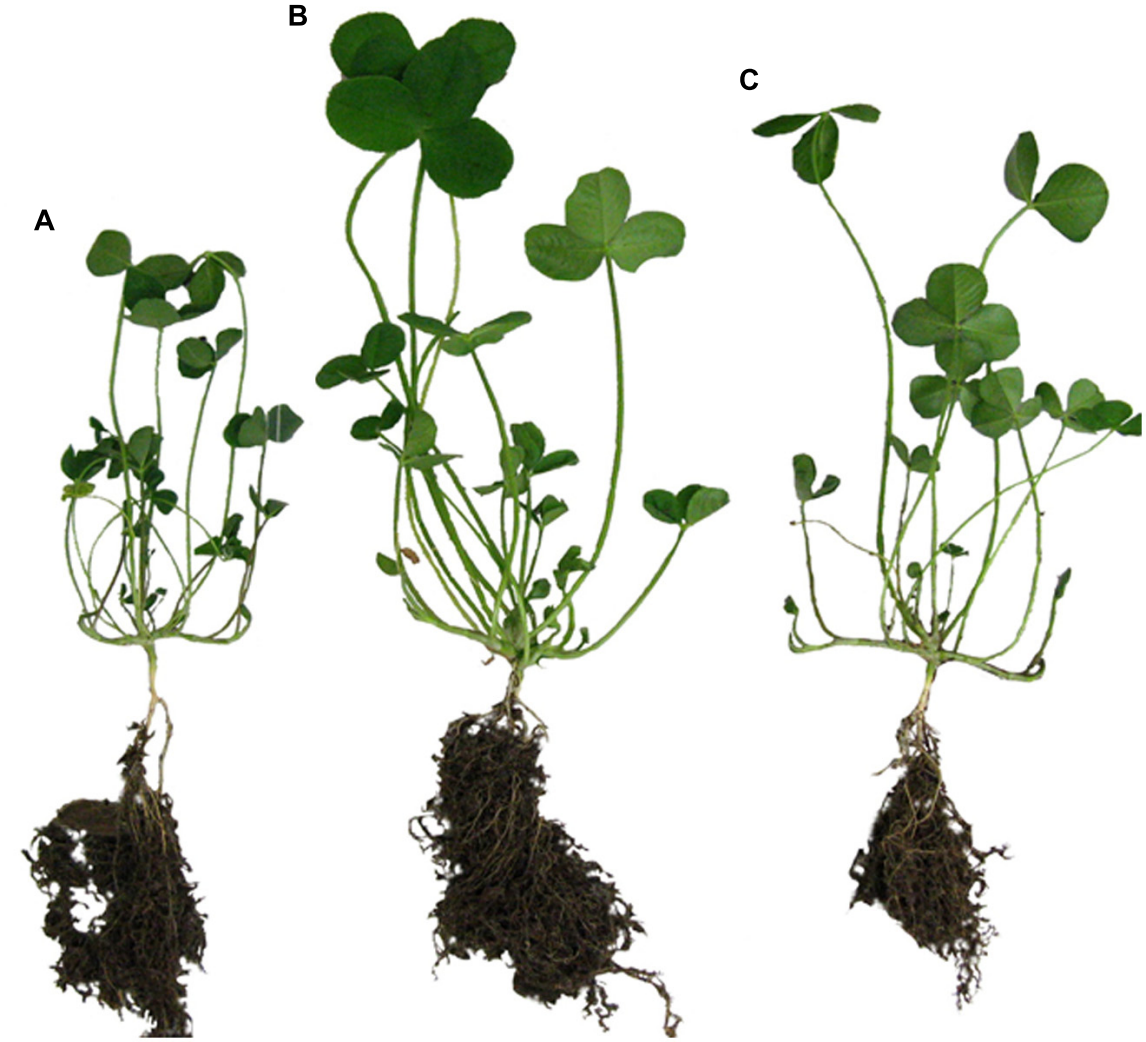
**Plant growth promoting effect of *C. botulinum* strain 2301 and *C. sporogenes* strain 1739 on clover after 4 weeks of growth inside a phytochamber within a natural soil system (pot experiments) using commercially available soil composed of natural clay, peat, and sod peat containing abundant nutrients. (A)** Inoculation with *Rhizobium leguminosarum* DSM 6039; **(B)** inoculation with *C. botulinum* strain 2301 and *R. leguminosarum* DSM 6039; **(C)** inoculation with *C. sporogenes* strain 1739 and *R. leguminosarum* DSM 6039.

For inoculation, spores of *C. sporogenes* strain 1739 or *C. botulinum* strain 2301 (10^5^–10^7^ spores ml^-1^ per plant) were applied together with *R. leguminosarum* DSM 6039 (1 × 10^7^ CFU ml^-1^ per plant) onto roots of clover seedlings. Like in the field trials (**Figures 1** and **2**), *C. botulinum* strain 2301 enhanced the growth of clover plants in the growth chamber experiment. Compared to negative controls, the *C. botulinum* strain 2301 treated plants showed a better growth index with increased branching of roots (**Figure 3**).

The plant growth promoting effect of *C. botulinum* and *C. sporogenes* could be observed both on root and shoot parts. The root system was larger, the roots were longer and the number of roots was increased. The plants were taller, the leaves were larger and the plants appeared all in all greener and more vital (**Figure 3**).

**FIGURE 4:**
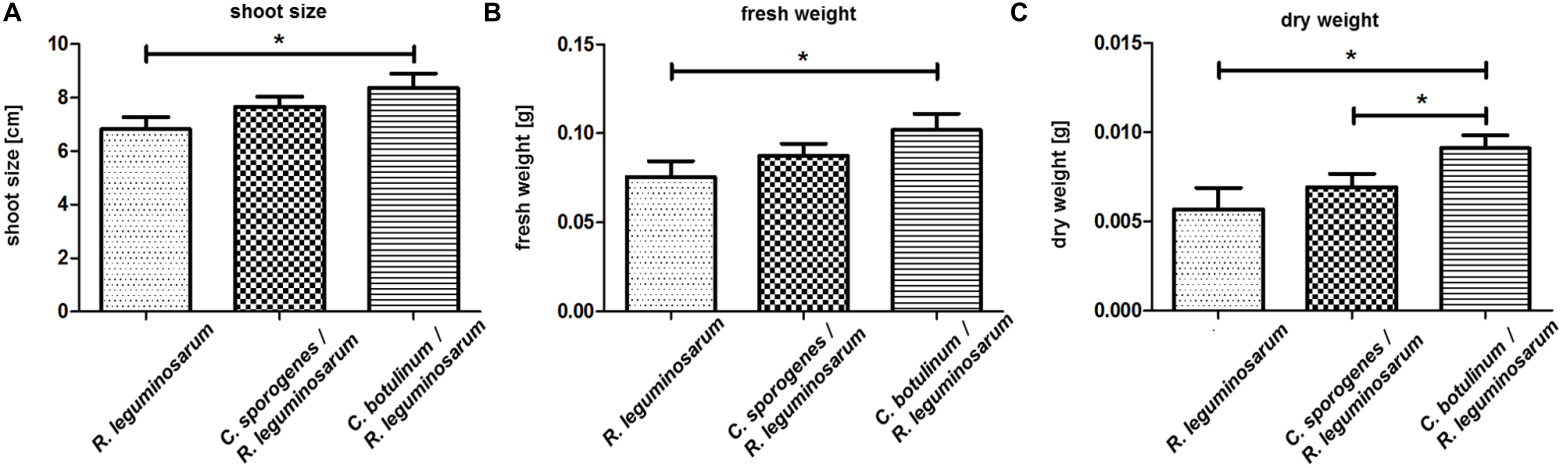
**Plant growth promoting effect of *C. botulinum* strain 2301 and *C. sporogenes* strain 1739 on clover after 4 weeks of growth in a natural soil system within a phytochamber.** Plants were inoculated with spores of *Clostridia* (10^6^) together with *R. leguminosarum* DSM 6039 (10^7^). The data were analyzed using students *t*-test and significance was assumed at ^∗^*p* < 0.05. **(A)** Shoot size (cm): *R. leguminosarum* DSM 6039: Mean ± SEM: 6,808 ± 0,465 (*N* = 24). *R. leguminosarum* DSM 6039 and *C. botulinum* strain 2301: Mean ± SEM: 8,355 ± 0,540 (*N* = 20). *R. leguminosarum* DSM 6039 and *C. sporogenes* strain 1739: Mean ± SEM: 7,662 ± 0,375 (*N* = 21). *R. leguminosarum* DSM 6039/*R. leguminosarum* DSM 6039 and *C. botulinum* strain 2301: *p* = 0,035. **(B)** Fresh weight (g): *R. leguminosarum* DSM 6039: Mean ± SEM: 0,076 ± 0,009 (*N* = 24). *R. leguminosarum* DSM 6039 and *C. botulinum* strain 2301: Mean ± SEM: 0,102 ± 0,009 (*N* = 20). *R. leguminosarum* DSM 6039 and *C. sporogenes* strain 1739: Mean ± SEM: 0,087 ± 0,007 (*N* = 21). *R. leguminosarum* DSM 6039/*R. leguminosarum* DSM 6039 and *C. botulinum* strain 2301: *p* = 0,046. **(C)** Dry weight (g): *R. leguminosarum* DSM 6039: Mean ± SEM: 0,006 ± 0,001 (*N* = 21). *R. leguminosarum* DSM 6039 and *C. botulinum* strain 2301: Mean ± SEM: 0,009 ± 0,001 (*N* = 17). *R. leguminosarum* DSM 6039 and *C. sporogenes* strain 1739: Mean ± SEM: 0,007 ± 0,001 (*N* = 18). *R. leguminosarum* DSM 6039 and *C. sporogenes* strain 1739/*R. leguminosarum* DSM 6039 and *C. botulinum* strain 2301: *p* = 0,046; *R. leguminosarum* DSM 6039/*R. leguminosarum* DSM 6039 and *C. botulinum* strain 2301: *p* = 0,028.

Clover plants showed significant differences between the *R. leguminosarum* DSM 6039 inoculated control plants and the plants inoculated additionally with *C. botulinum* strain 2301 (**Figure 4**).

**Figure 4** shows significant differences (*p* < 0.05) between the *R. leguminosarum* DSM 6039 (10^7^) inoculated and additionally *C. botulinum* strain 2301 (10^6^) inoculated plants reflecting the plant growth promoting effect of *C. botulinum* strain 2301 on clover after 4 weeks of growth in soil within a phytochamber. A better growth reflected by a higher shoot size, fresh, and dry weight yielding a higher biomass (**Figure 4**) could be demonstrated.

### *In planta* Detection of *C. botulinum* Strain 2301 by PCR based Analysis

**FIGURE 5:**
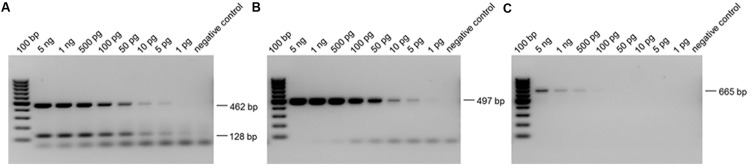
**Detection limits of PCR experiments. (A)** PCR with IBTSB F2/IBTSB R2 (462 bp, BoNT D) and IBTSB F4/IBTSB R4 (128 bp, BoNT D): detection limit 5 pg; **(B)** PCR with DS11/DS22 (497 bp, BoNT D): detection limit 5 pg; **(C)** PCR with P930/P932 (665 bp, 16S-rDNA): detection limit 100 pg.

The detection of the inoculated *Clostridia* by crushing the plant material with liquid nitrogen and subsequent DNA isolation without further enrichment produced in variable results (not shown). Therefore, plant samples were washed in PBS and transferred to selective media (RCM) for enrichment of *Clostridia*. After 24 h of growth, enrichment cultures were subjected to DNA isolation. The resulting total DNA contained, compared to the above mentioned liquid nitrogen extraction, a significantly higher percentage of bacterial DNA. The sensitivity of PCRs was similar to those reported previously (Takeshi et al., 1996) and 5 pg of template DNA (approximately 1650 cells) could be detected by staining their amplified products on agarose gels. **Figure 5** demonstrates the sensitivity and specificity of the detection method.

As little as 5 pg of template DNA from toxigenic *C. botulinum* strain 2301 could be detected by the BoNT/CD multiplex PCR system (**Figure 5A**) and primer pair DS11/DS22 (**Figure 5B**) respectively. The lowest amount of template DNA of type D, which produced observable products on agarose gels with primer pair P930/P932, was 100 pg (**Figure 5C**). The amplification products were sequenced to proof correct amplification, because database search showed, that some regions of plants chloroplasts and mitochondria have only one mismatch in the target region of the used primer.

**FIGURE 6:**
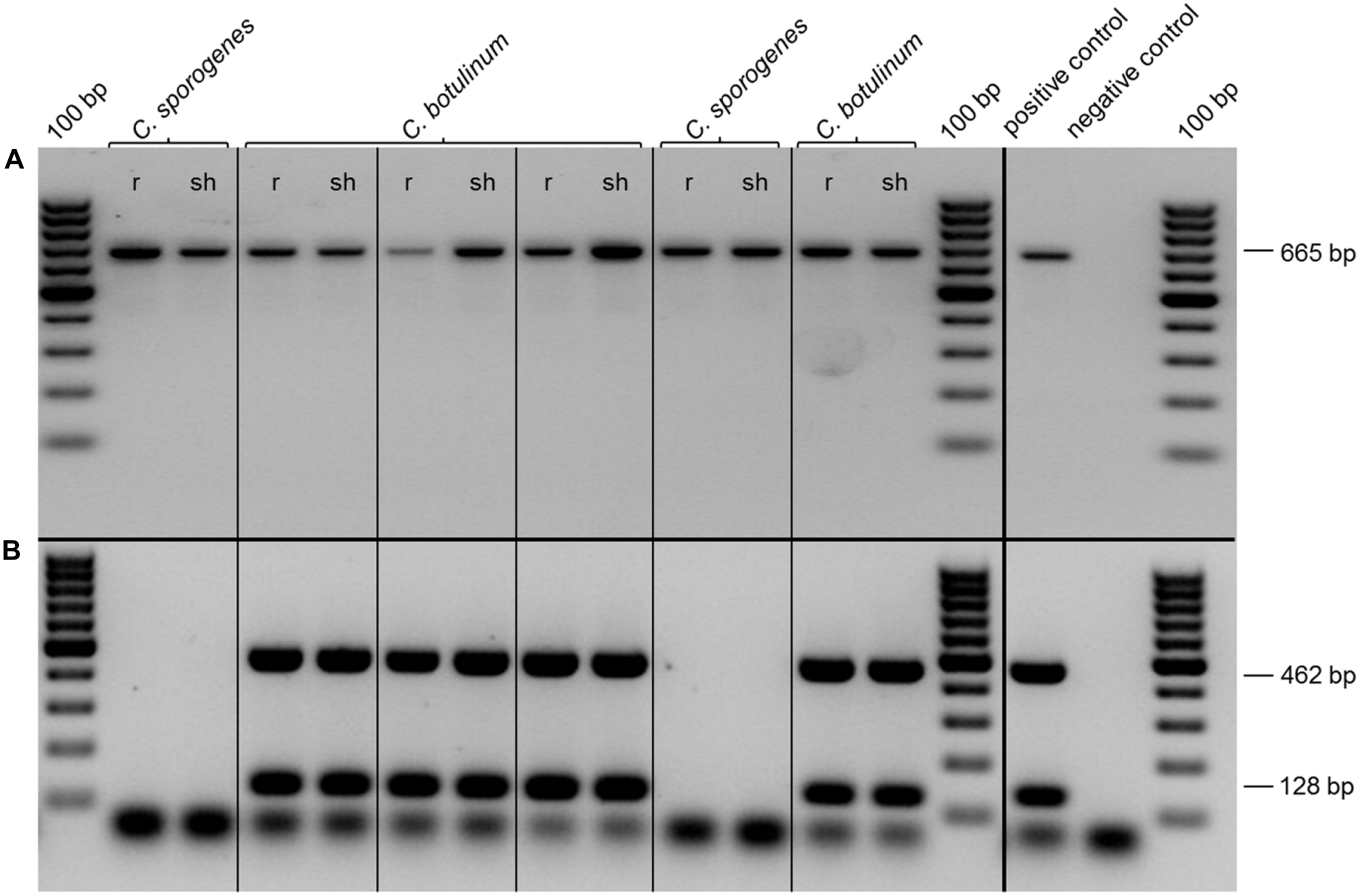
**Polymerase chain reaction detection of *Clostridia* in root (r) and shoot (sh) of inoculated plants. (A,B)** PCR with 10 ng of DNA isolated from root (r) and shoot (sh) after axenic growth. **(A)** PCR with P930/P932 (665 bp, 16S-rDNA). **(B)** PCR with F2/R2 (462 bp, BoNT D) and F4/R4 (128 bp, BoNT D).

**FIGURE 7:**
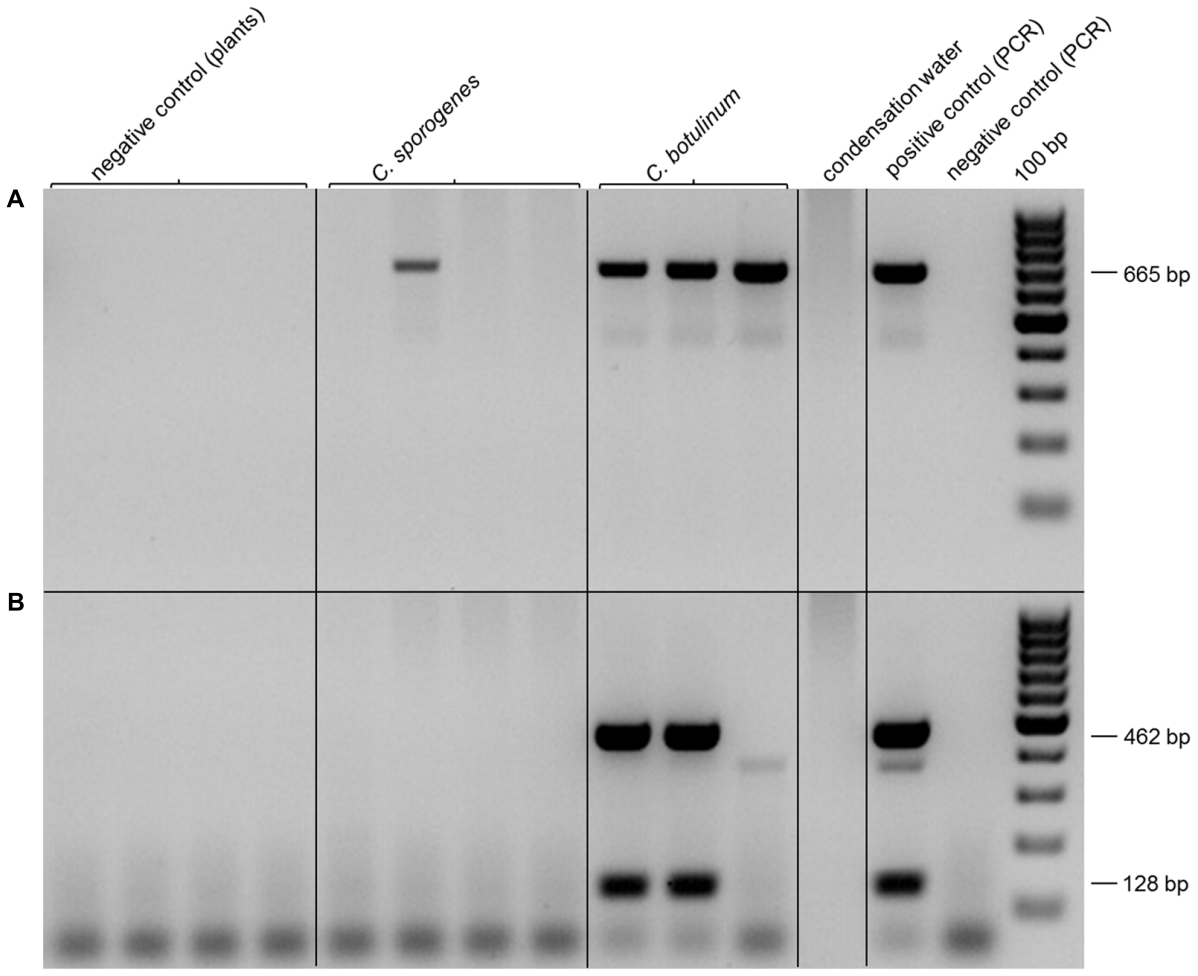
**Polymerase chain reaction Detection of *Clostridia* in shoots of inoculated plants. (A,B)** PCR with 10 ng of DNA isolated from shoot after growth in soil. **(A)** PCR with P930/P932 (665 bp, 16S-rDNA). **(B)** PCR with F2/R2 (462 bp, BoNT D) and F4/R4 (128 bp, BoNT D).

This approach allowed a qualitative but no quantitative detection. By the use of a PCR system with DNA isolated from enrichment cultures of inoculated plants, *C. botulinum* strain 2301 could be detected sensitively in roots (r) and in shoots (sh) of clover grown for 4 weeks in an axenic system (**Figure 6**), as well as in the shoots of clover after 4 weeks of growth in a soil system (**Figure 7**). Identical results for the detection of *C. botulinum* strain 2301 were achieved with both primer sets.

### Localization of *C. botulinum* Strain 2301 by FISH/CLSM Analysis

To determine the colonization behavior of the used strains on clover plants *in situ*, EtOH- and PFA- fixed root samples, from the experiments described above, were analyzed applying FISH in combination with CLSM. In addition, samples from the same plants were taken for further DNA isolations which were also subjected to PCR experiments. The fixed root samples were hybridized with species- and domain specific oligonucleotide probes.

**FIGURE 8:**
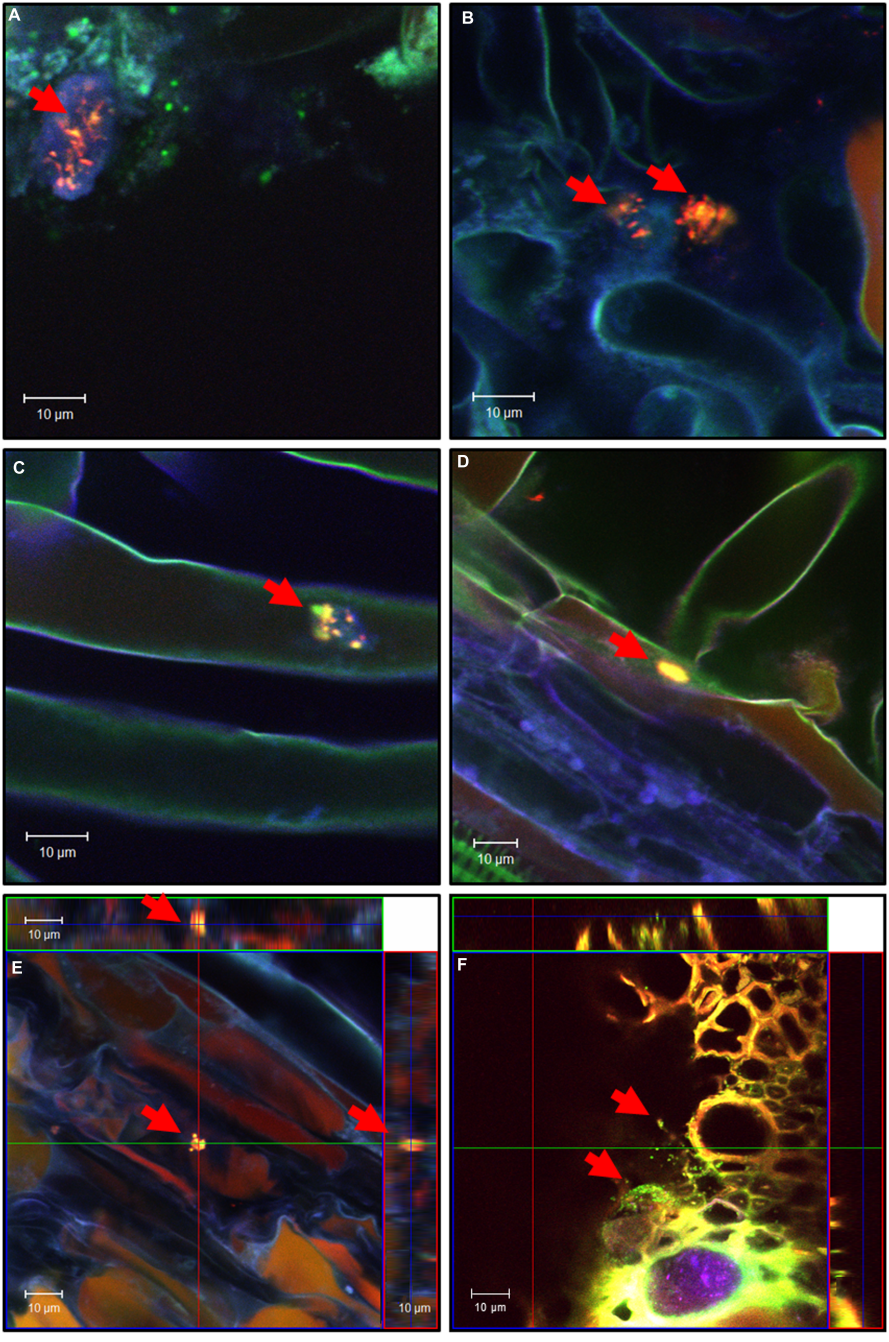
**Confocal images of sections of fixed white clover roots, inoculated with *R. leguminosarum* DSM 6039 and spores of *C. botulinum* strain 2301, after 4 weeks of growth in axenic system.** For *in situ* hybridization probe Chis150-Cy3 and EUB338-I,-II,-III-Fluorescein was used. *Clostridia* of subcluster I are detectable by a yellow fluorescence signal in the rgb-image (red arrows). **(A–E)** Longitudinal roots sectioning; **(F)** lateral root section. **(E,F)** Orthogonal view of a three-dimensional confocal image generated from a z-stack of xy scans.

**Figure 8** shows confocal images of sections of fixed root samples of white clover after 4 weeks of growth in an axenic system. Using FISH (Chis150-Cy3, EUB338-I,-II,-III-Fluorescein) analysis in combination with CLSM, the colonization behavior could be analyzed in detail. Within this experiment, *R. leguminosarum* DSM 6039 and spores of *C. botulinum* strain 2301 were co-inoculated. Detailed microscopic analyses of the colonization behavior demonstrate that cells of *C. botulinum* strain 2301 could be mainly detected epiphytically (**Figures 8A–D**) on root surfaces in the rhizosphere of clover, but also occasionally as endophytes (**Figures 8E,F**). By producing three-dimensional orthogonal images of z-stacks from xy scans of optical sections, precise localization of the colonizing cells was possible. *Clostridia* were found in the intercellular spaces of the root cortex of clover plants (**Figure 8F**). The bacteria primarily colonize lateral roots. In general, the cell numbers of recognized *Clostridia* were low. The highest density of colonization was observed where root hairs emerged from the main root and *C. botulinum* strain 2301 shows colonization behavior in single cells and larger microcolony-like clusters of cells consisting of *Clostridia* and other bacteria.

It could be demonstrated that both pathogenic and non-pathogenic *Clostridium* strains, colonize clover and can also endophytically persist in plant tissues to some extent and that there was no apparent difference between the colonization pattern of *C. botulinum* strain 2301 and *C. sporogenes* strain 1739.

The results of specific PCR based detection methods and *in situ* localization studies (FISH/CLSM studies), reported in this communication, demonstrate a systemic colonization of clover plants by *C. botulinum*.

## Discussion

### Specific Detection of *C. botulinum* Strains *In planta*

Using PCR based molecular detection methods with different primer pairs targeting the BoNT D gene encoding botulinum neurotoxin type D as well as a FISH/CLSM approach it was possible to specifically detect *C. botulinum* within the plant material, which shows that *Clostridia* can spread from primary inoculated roots to above ground shoot parts (**Figures 6** and **7**). Enrichment of *C. botulinum* in harvested plant material prior to DNA isolation ensured a reliable, sensitive, non-quantitative detection of *C. botulinum* in plant material. In the case of non-sterile soil as growth substrate, inoculated *Clostridia* were exposed to naturally occurring microbiota affecting survival and fitness of bacteria in the rhizosphere and therefore efficiency of colonization was much lower. This was also shown, e.g., for the colonization of *Salmonella enterica* in soil grown lettuce plants as compared to axenic plants (Hofmann et al., 2014).

Using cultivation-dependent methods, the true composition and abundance of different microbes is drastically altered (Torsvik et al., 1990). While targeted enrichment of specific microbes from environmental samples improved the detection limit drastically, it is not applicable for accurate localization and only a qualitative statement about the occurrence of microorganisms can be achieved (Wagner et al., 1993). Therefore, *in situ* detection and localization of inoculated *Clostridia* in plant material using *in situ* labeling (FISH) with specific oligonucleotide probes in combination with CLSM was used. An endophytic spreading of non-pathogenic (*C. sporogenes*) and pathogenic (*C. botulinum*) strains to rhizodermis layers, inner root cortex and to the shoot of clover could be demonstrated (**Figure 8**). The observed endophytic colonization of clover roots and the persistence of colonization of plant tissues and furthermore the evidence for plant growth promotion without any deleterious effect to the host plant obviously reflects a mutual beneficial relationship between *C. botulinum* and clover. Highest colonization rates were found in the root hair zone presumably due the high available surface area for colonization. *C. botulinum* showed predominantly a colonization behavior in single cells and not, as known from other organisms, in large cell clusters. Occasionally, however, also small clusters of cells, consisting of *Clostridia* and other bacterial strains reminiscent of microcolonies or biofilm structure could be observed.

One reason for detecting low numbers of clostridial cells by FISH-analysis is the low physiological activity of cells in resting stages and spores with a low content of ribosomes and a poor accessibility for probes. The amount of rRNA in cells is directly correlated with conferred fluorescence of probes directed to rRNA (DeLong et al., 1989) and therefore a low physiological activity leads to weak or not detectable signals. This might have been the reason for the difficult detection of *Clostridia* cells also *in planta*. Another challenge in microscopic analysis of soil or plant associated microorganism with fluorescent probes is the enormous autofluorescence of plant material or mineral soil particles (Hahn et al., 1992). In plants grown in soil the detection of inoculated *Clostridia* within the plant material was more complicated and *C. botulinum* strain 2301 could not be clearly found in all samples tested. Because the used FISH probe (Chis-150) detects *Clostridia* only on genus level, discrimination between naturally occurring and inoculated strains is not possible. This constitutes a difficulty for a reliable detection of these cells using this approach in a complex, soil based plant system. Therefore, the PCR detection of the neurotoxin D encoding gene is the most reliable method for a safe detection of BoNT D producing Clostridia cells in a complex system.

### Systemic Spreading of *C. botulinum*

A prerequisite of systemic spreading of bacteria in plants is the penetration of the inner pith of root cortex to reach vascular tissues and the vascular xylem system, where the water transport toward aerial parts takes place. Alternatively, also active movement and colonization by *Clostridia* is possible. The clostridial cells could be detected and localized endophytically in plant tissues (**Figure 8**), but it was not possible to distinguish if *Clostridia* were present for example in xylem or phloem compartments of plant roots. As anaerobic bacteria, *Clostridia* were detected in tissue compartments with reduced oxygen concentration. Endophytic colonization by nitrogen fixing *Clostridia* has also been previously described in *Miscanthus* plants (Minamisawa et al., 2004; Miyamoto et al., 2004; Ye et al., 2005). First, these bacteria colonized lateral root junctions, using these sites to enter the root cortex and to finally infect the whole plant by colonizing the xylem. Invasion of *Medicago truncatula* and *M. sativa* by enteric bacteria was demonstrated by Dong et al. (2003) and extensive colonization of lateral root cracks by enteric bacteria, similar to the colonization by nitrogen fixing endophytes, leads to entry of the plant.

### Plant Growth Promoting Effect

Plant growth promoting effects have been detected with a wide variety of PGPB (plant growth promoting bacteria). These can be based on different or even multiple mechanisms, like the production of phytohormones, e.g., auxin production, which stimulate the proliferation of the root system improving the uptake of limiting nutrients (Franche et al., 2008; Richardson et al., 2009). In addition, the acquisition of nutrients with low solubility, like phosphate or ferric iron (Jin et al., 2006), was increased by PGPB. The observed plant responses of clover after inoculation with *C. botulinum* strain 2301 could be due to an alteration of the phytohormone status, which needs to be studied further on in more detail. However, in axenic plants no significant difference in growth was found after inoculation. This may be due to oxic conditions in MS medium and to unfavorable environmental conditions for the anaerobic bacteria, like *Clostridia*, which do not allow proliferation of vegetative cells or spore germination and finally efficient root colonization. In contrast, in the soil system clover plants showed a significantly higher growth if inoculated with *C. botulinum* strain 2301 compared to control plants that were inoculated with *Rhizobia* alone. In non-axenic conditions, the presence of other microorganisms probably created a low oxygen environment in the rhizosphere and on the root surface favoring also the colonization of roots by *Clostridia* (Minamisawa et al., 2004).

### Plant Associated Toxin Producing Bacteria and Pathogens as Health Threat

A better understanding of microbe plant interactions will enable the development of evidence based policies, procedures and technologies to reduce the risk of contamination of fresh food products (Berger et al., 2010). As demonstrated in this work, toxin producing clostridial strains like *C. botulinum* strain 2301 not only successfully colonize the rhizosphere but also endophytically colonize clover roots leading to systemic spreading within the whole plant. The consumption of these infected plants could result in serious health risks for grazing animals such as cows and horses. It throws an interesting light also on the observation that horses grazing on pastures with high clover content are exposed to a higher risk for Equine Grass Sickness, a dysautonomia with *C. botulinum* etiology (Böhnel et al., 2003). Remarkably, the prevalence of botulism, determined by the detection of BoNT genes in fecal samples of cattle and horses, was higher when animals were on a pasture compared to when they were kept inside a barn (Steinman et al., 2006).

An endophytic colonization of wild rice species and of pioneer plants (*M. sinensis, S. spontaneum*) with nitrogen fixing *Clostridia* has been demonstrated (Minamisawa et al., 2004; Miyamoto et al., 2004; Ye et al., 2005), similar as with *C. botulinum* strain 2301 and clover. For a better understanding of the mechanisms of these bacteria–plant interactions, further studies are needed, because although *Clostridia* are distributed in, e.g., soil, their lifestyle as plant associated or endophytic bacteria has not been understood in detail so far. The nature of the consistently observed plant growth promotion effect of *C. botulinum* strain 2301 and *C. sporogenes* strain 1739 strains also awaits further investigation. Although *Clostridia* are generally characterized by an anaerobic lifestyle, our results clearly demonstrates that certain *Clostridia* can successfully colonize plants and are also able to persist and proliferate within plants. The availability of knowledge about the route of infection and critical plant and microbial factors that influence the colonization efficiency of plants by human pathogenic or toxigenic bacteria is important for the design of prevention strategies in order to guarantee the safety of foods for animal and mankind (Dressler and Saberi, 2009). In a recent study deceased cows, farmers and family members as well as feed and house dust were analyzed for *C. botulinum* and four of the examined people showed symptoms of visceral botulism and *C. botulinum* antigens were detected (Dressler and Saberi, 2009; Krüger et al., 2012). Thus, it seems to be of utmost importance to intervene with the very early process of spreading of *C. botulinum* in the farm environment which appears to be initially dependent on the colonization of fodder plants by *C. botulinum*.

## Author Contributions

MZ: performing experiments, conducting the work, design of the work, analysis, interpretation of data for the work, responsible for the integrity of the work as a whole, final approval of the version to be published.

MS: design of the work, acquisition, analysis, interpretation of data for the work, critically revising, final approval of the version to be published, ensuring that questions related to the accuracy or integrity of any part of the work are appropriately investigated and resolved, responsible for the integrity of the work as a whole.

MR: analysis, interpretation of data for the work, critically revising, final approval of the version to be published, ensuring that questions related to the accuracy or integrity of any part of the work are appropriately investigated and resolved.

AI: design of the work, analysis.

FG: design of the work, critically revising, final approval of the version to be published.

HB: design of the work, critically revising, final approval of the version to be published.

AH: design of the work, acquisition, analysis, interpretation of data for the work, critically revising, final approval of the version to be published, ensuring that questions related to the accuracy or integrity of any part of the work are appropriately investigated and resolved, responsible for the integrity of the work as a whole.

## Conflict of Interest Statement

The authors declare that the research was conducted in the absence of any commercial or financial relationships that could be construed as a potential conflict of interest.
